# The Book World of Medicine and Science

**Published:** 1903-07-18

**Authors:** 


### The Book World of Medicine and Science.

The Manual Treatment of Diseases of Women. Gustaf Norström, M.D., Stockholm. (New York and London: G. E. Stechost. 8vo. Pp. 230. 1903. 10s. net.)

This volume is a defence of the practice of intra-pelvic massage, usually associated with the name of Thure Brandt, and is a specimen of *ex parte* pleading not calculated to advance its object very greatly. The various forms of "Swedish massage," as the public terms it, have brought themselves a little under suspicion in the Anglo-Saxon medical world. The excellent therapeutic gymnastics evolved and systematised by Ling and his followers produced a school of professors of the art whose enthusiasm outran their discretion. Every debility and every ailment were to be cured by Swedish movements and Swedish massage. Thure Brandt, a non-commissioned officer in the Scandinavian Army began intra-pelvic manipulations as a panacea for the common pelvic ailments of women, about 1871, and we have seen in 1902 ethmoiditis treated by "Swedish massage." We are unable to say what intermediate conditions of the abdominal or thoracic organs have not been similarly treated. The above summary may seem hard but it is perfectly true, and is the best reply to the complaint which Dr. Norström makes, that intra-pelvic massage has been received with hesitation and criticism in France, England and America. It is not necessary at this date to discuss the question whether intra-pelvic massage is repugnant to the feelings of female patients of any particular nationality, or to set up a querulous plea that Scandinavian ladies are not less modest than those of the rest of Europe or America; no one who had ever visited Norway or Sweden could doubt it. Every gynæcologist knows that new methods of exploration and treatment demand a gradual local or national training, to be directed through the everyday practice of the medical men of the nation or area involved, before the unhappy subjects of such practice become accustomed to their use. To make a special attack as Dr. Norström does, on American gynæcologists (or on American women) for rejecting the results of this form of massage indicates lack of reading on the author's part. One of the best summaries of the subject we know is by Emmett in Vol. I. of Keating and Coe's "Clinical Gynæcology." In fact the student of the ethics, the application, and the limitations of this method will learn more from Dr. Emmett's article than he will from the volume before us. After carefully reading the volume and some recent criticisms on the subject we see no reason to modify the opinions formed some years ago, that intra-pelvic massage is not a method to be applied without grave reason; that it should preferably be administered by female masseuses, for we deny at once that masseuses are not to be found strong enough to administer the course. If, as the author states, the work of massage of this especial kind is so hard that only men can apply it, there is no question that powerful women could apply it for a limited number of cases each day. The value of intra-pelvic massage is, we believe, limited to cases of perimetric effusion and adhesion; further that great care in the diagnosis of cases must be taken, or that, if such care is not taken, grave calamities may occur; ovarian cysts, pyosalpinx, ectopic gestation, tubercle of the genital tract, malignant growths, cysts of the broad ligament, chronic hæmatocele are all conditions to be strictly avoided when massage is proposed. And massage should never be attempted in any acute disease or exacerbation of a pre-existing disease. This list alone is enough to make the wise man pause. "One can scarcely over-estimate the amount of discretion which it is essential to exercise in utilising this valuable aid to our treatment of the various pelvic diseases of women." These are the words of a well-known gynæcologist in a well-known American text-book. They should be pondered before accepting with too great faith the opinions of Dr. Norström.

Chronic Headache and its Treatment by Massage. By Gustaf Norström, M.D., Stichert. (New York. 1902. 8vo. 4s. 6d. Pp. 59.)

This brochure is too long for a pamphlet and too small for a book. It is one of the many evil effects of cheap printing; it would have made a useful little paper at a medical society or contribution to a clinical journal. The practical idea of the author is sound, that many cases of neuralgia of the scalp, especially the vertical and posterior scalp, are due to changes in and around the sheaths of the posterior, cervical, and occipital groups of nerves, and to chronic myositis of the muscles of the neck. The corollary is also obvious that many of these conditions can be relieved by massage.

Disinfectants and Antiseptics. By E. T. Wilson, M.B.Oxon., F.R.C.P.Lond. (Pp. 4. Card. Price 1s. per dozen).

This very excellent card of directions for the use of disinfectants should find a place in every household. The fact that practically all chemicals which have bactericidal properties are also poisonous to man, if taken in sufficient quantity or concentration, is perhaps the reason why their use is not more general among the lay public. But with this card to guide him no one need have fear on this score. Many useful hints are given as to what antiseptics will stain linen or corrode metals and so forth, and the best one to select for any given purpose may readily be ascertained by a glance at the directions given. Some general advice is appended on the precautions to be taken, when an infectious disease has broken out in a house, to prevent its spreading to other members of the household, and this ought to prove most valuable. The card should have a large sale proportionate to its usefulness among the public.

